# MFN2 suppresses cancer progression through inhibition of mTORC2/Akt signaling

**DOI:** 10.1038/srep41718

**Published:** 2017-02-08

**Authors:** Ke Xu, Guo Chen, Xiaobo Li, Xiaoqin Wu, Zhijie Chang, Jianhua Xu, Yu Zhu, Peihao Yin, Xin Liang, Lei Dong

**Affiliations:** 1 Central laboratory, General Surgery, Putuo Hospital, and Interventional Cancer Institute of Chinese Integrative Medicine, Shanghai University of Traditional Chinese Medicine, 164 Lanxi Rd, Shanghai 200062, PR China; 2 Winship Cancer Institute, Emory University School of Medicine, Atlanta, GA 30322, USA; 3 Tianjin Key Laboratory of Molecular Design and Drug Discovery, Tianjin Institute of Pharmaceutical Research, Tianjin 300193, China; 4 School of Pharmacy, Institute for Liver Diseases of Anhui Medical University, ILDAMU, Key Laboratory of Anti-inflammatory and Immune Medicine, Anhui Medical University, Hefei, 230032, China; 5 State Key Laboratory of Membrane Biology, School of Medicine, School of Life Sciences, Tsinghua University, Beijing 100084, China; 6 Department of Clinical Laboratory, Tianjin Huanhu Hospital, Tianjin Key Laboratory of Cerebral Vessels and Neural Degeneration, Tianjin 300350, China; 7 State Key Laboratory of Bioreactor Engineering & Shanghai Key Laboratory of New drug design, School of pharmacy, East China University of Science and Technology, 130 Meilong Rd, Shanghai 200237, PR China; 8 Department of Pediatrics, Division of Hematology/Oncology, Aflac Cancer and Blood Disorders Center, Children’s Healthcare of Atlanta, Emory University School of Medicine, Atlanta, GA 30322, USA

## Abstract

The mitochondrial GTPase mitofusin-2 (MFN2) has previously been reported to play a role in regulating cell proliferation, apoptosis and differentiation in a number of cell types. Here, we report that breast cancer patients with low MFN2 expression are associated with poor prognosis as compared to patients with high MFN2 expression. We find that MFN2 knockout from MCF7 and A549 cells via Crispr/Cas9 greatly promotes cell viability, colony formation, and invasion of cancer cells *in vitro* and *in vivo*, which were confirmed by colony formation assay, transwell invasion assay, and tumor xenograft model. Signaling analyses suggest the mammalian target of rapamycin complex 2 (mTORC2)/Akt signaling pathway is highly elevated in MFN2 knockout cancer cells. The elevated mTORC2 promotes cancer cell growth and metastasis via Akt^S437^ phosphorylation mediated signaling pathway. Mechanistic studies reveal that MFN2 suppresses mTORC2 through direct interaction by binding its domain HR1. Inhibition of mTORC2 significantly suppresses MFN2 deficient tumor growth. Collectively, this study provides novel insights into the tumor progression associated with MFN2 deficiency and suggests that the importance of mTORC2 inhibitor in the treatment of MFN2 downregulated cancer patients.

The mitochondrial GTPase mitofusin-2 (MFN2) gene, also called the hyperplasia suppressor gene (HSG), was originally found in vascular smooth muscle cells^1^. MFN2 localizes to the mitochondrial outer membrane and plays an essential role in mitochondrial fusion, cardiac metabolism, and mitochondria mediated apoptotic pathway^2^,^3^,^4^. Previous studies have indicated that MFN2 notably suppresses vascular smooth muscle cell growth and proliferation via the inhibition of the RAS-ERK MAPK signaling pathway^5^. Recently, several studies have investigated the function of MFN2 in various malignancies, including hepatocellular, urinary bladder and gastric cancers^6^,^7^,^8^. Clinical and epidemiological evidence revealed that low MFN2 expression in many types of cancer is associated with poor prognosis^9^. However, the underlying mechanism by which MFN2 exerts its antitumor effects remains unclear. Therefore, exploration of MFN2 function will help us to understand its role in the pathogenesis and treatment of various tumors.

Mammalian target of rapamycin (mTOR), a threonine/serine kinase evolutionarily conserved in all eukaryotic cells, is considered to be the master coordinator of extracellular signals, an integration point that regulates cell growth, metabolism and proliferation in an appropriate and finely tuned response^10^,^11^,^12^. mTOR nucleates two distinct multiprotein complexes known as mTOR complex 1 (mTORC1) and mTOR complex 2 (mTORC2), characterized by the presence of raptor and Rictor respectively. mTORC1 regulates cell growth and proliferation via S6 kinase 1 (S6K1) and eIF-4E-binding protein-1 (4E-BP1), both key regulators of protein biosynthesis^13^. mTORC2 comprises mTOR, Rictor, mammalian lethal with SEC13 protein 8 (mLST8), stress-activated protein kinase (SAPK)-interacting protein (Sin1), and protor, and phosphorylates AGC kinases, such as Akt, serum/glucocorticoid-regulated kinase 1 (SGK1), and PKC, all of which are associated with cancer^14^,^15^. mTORC2 phosphorylates the hydrophobic motif site Ser473 on the Ser/Thr kinase Akt as well as the stability of Akt that is necessary for its activation^16^,^17^. Thus, mTORC2 is involved in a variety of processes that are regulated by Akt, including cell survival, glucose metabolism, and cellular differentiation^18^,^19^.

In most cancers, the mTOR complexes are “hijacked” by hyper-activation as a result of upstream events^20^. The aberrant activation can occur by the abnormal oncogenic activity of critical proteins and/or within other inactivation of tumor suppressor proteins. Gene mutations resulting in oncogenic activation of receptors such as EGFR and HER2 over activate mTOR in several cancers, such as breast, lung and gastric cancers^21^,^22^. Mutated Akt, RAS and RAF which are present in a variety of tumors, such as ovarian, thyroid and pancreatic cancers, have also been identified to cause hyperactivation of mTOR^23^,^24^,^25^. On the contrary, the mutated tumor suppressor PTEN, which is inactivated in a significant proportion of endometrial cancers, glioblastomas and melanomas, is reported to be essential for deactivation of the PI3K-mTOR pathways^26^,^27^.

Here we identify MFN2 as a mTORC2-interacting protein. We find that MFN2 inhibits mTORC2 kinase activity and also the activation of Akt. Furthermore, we show that MFN2 negatively regulates cancer cell survival through inhibiting mTORC2 and Akt. These findings reveal MFN2 as a regulator of mTORC2 signaling via Akt^S437^ phosphorylation mediated signaling pathway.

## Materials

### Cell lines and reagents

Human breast cancer cell lines MCF-7 and human NSCLC cell line A549 (obtained from the Cell Bank of Chinese Academy of Science) were maintained in RPMI 1640 medium or DMEM/F12 medium (Gibco Industries, Inc.) with 10% fetal bovine serum (Gibco Industries, Inc.) at 37 °C in a humidified atmosphere with 5% CO_2_. MFN2 overexpression plasmid (Flag-MFN2) and Rictor overexpression plasmid (Myc-Rictor) were purchased from Addgene (Cambridge, MA). MCF-7 and A549 were transfected using Lipofectamine 3000 (Thermo Fisher Scientific, Rockford, IL) according to the manufacturer’s instruction.

Primary antibody MFN2, Akt, p-Akt (S473), p-Akt (S308), mTOR, p-mTOR (S2481) and p-mTOR (S2448) were purchased from Cell Signaling Technology, ERK, p-ERK, STAT3, p-STAT3, myc, flag, Rictor and β-actin were purchased from Santa Cruz Biotechnology. Palomid 529 (P529) was purchased from Selleck Chemicals.

### Tissue samples

Human breast cancer samples were collected at the time of surgical resection at Putuo Hospital, Shanghai University of Traditional Chinese Medicine, P.R. China, from January 2008 to December 2010. Written informed consent was obtained from the patients, in accordance with the institutional guidelines, before sample collection, and the study was approved by the Committees for the Ethical Review of Research at the Putuo Hospital, Shanghai University of Traditional Chinese Medicine, P.R. China. The methods were performed in accordance with the approved guidelines. All patients had a histological diagnosis of breast cancer and received radical resection. None of the patients included in the study had received neoadjuvant therapy before surgery. Samples for further histopathological analysis were immediately snap frozen in liquid nitrogen and stored at −80 °C freezer.

### MFN2 knockout via Crispr/Cas9

Cells were transfected with MFN2 Crispr/Cas9 KO Plasmid (Santa Cruz, sc-400536) using lipofectamine 2000 (Invitrogene, Carlsbad, CA) according to the manufacturer’s instructions. Forty-eight hours after transfection, the GFP expressing cells were sorted by flow cytometry into 96-well plate at density of 1 cell per well. MFN2 deletion from each single clone was then confirmed by Western blot.

### Colony formation assay

Parental or MFN2 knockout cells (800 cells/well) were plated into the 6-well plate and cultured for 7–10 days. Cells on the plates were then fixed and stained with 0.1% crystal violet in 20% methanol. Surviving colonies were counted and the surviving percentage was calculated and normalized with parental cells. For soft agar colony formation, 2 × DMEM containing 20% FBS, 200 U/ml penicillin, 200 μg/ml streptomycin and 0.6% melted agarose were coated into 6-well plates, which was then incubated at room temperature for 30 min to allow the bottom layer to solidify. 1 × 10^4^ cells in 500 μl DMEM were mixed with 500 μl 2 × DMEM containing 20% FBS and 500 μl of 1.2% agar, and then placed on the bottom layer. The plates were incubated at 37 °C with 5% CO_2_ for 3 weeks before counting.

### B16F10 melanoma-lung metastasis model

The B16F10 metastasis model *in vivo* was performed as described^28^. Briefly, C57BL/6J mice were intravenously injected with 10^6^ parental or MFN2 knockout B16F10cells. Mice were sacrificed at 14 days after cell injection and the lungs were then collected and examined for the presence of black metastatic foci.

### Transwell invasion assay

The cell invasion ability was examined using matrigel invasion Chambers (BD Bioscience, Woburn, MA, USA). For matrigel coating, matrigel diluted in cold PBS was added on the upper chambers and incubated for 1 hour at 37 °C for gel formation. Then, 10^5^ cells in serum-free medium were placed into the coated upper chamber. Complete medium with 10% FBS were added to the lower chamber. After 24 hours, the cells remaining on the upper membrane were removed with cotton wool, whereas the cells that had invaded through the membrane were stained with 20% methanol and 0.1% crystal violet and counted.

### Wound healing assay

Cells were seeded in 24-well plate and starved for 24 h before scratching with pipette tip. After scratching, cell were washed with PBS, photographed and placed into medium with 1% FBS to prevent dividing. After 24 hours, matched-pair wound regions were photographed. The areas of wound were calculated by image J.

### Western blot analysis

Proteins were resolved in an SDS/PAGE gel and subjected to immunoblot analysis using monoclonal antibodies. All antibodies were used at 1 mg/ml of working concentration in PBS with 5% dried-milk. The membrane was further probed with horseradish peroxidase (HRP)-conjugated rabbit anti-mouse IgG (Santa Cruz, 1:2000) or HRP-conjugated goat anti-rabbit IgG (abcam, 1:2000) and the protein bands were visualized using enhanced chemiluminescence (Amersham Pharmacia Corp, Piscataway, NJ). Quantification of protein bands was performed using the ImageJ software.

### Site-directed mutagenesis

MFN2 deletion mutants, including Δ1-400, and Δ1-600 were generated by inverse PCR using the flag-WT MFN2 as template. Sequences of 5′-phosphorylated primers were used for PCR as follows: Δ1-400, forward: 5′-CTG AAA TTT ATT GAC AAA CAG C-3′, reverse: 5′-GCA TCG AGA GAA GAG CAG GGA CAT-3′; Δ1-600, forward: 5′-GTT TCC ATG GTT ACC GGC CTG GCC-3′, reverse: 5′-GCA TCG AGA GAA GAG CAG GGA CAT-3′. After PCR, the amplification products were digested by DpnI to remove the non-mutated template and circularized by ligation, followed by transformation into DH5α for amplification. All mutants were confirmed by sequencing.

### Immunoprecipatation

Cells were collected in lysis buffer (50 mMTris-HCl pH 7.6–8.0, 0.5% NP-40, 10 mM NaF, 2 mM Na_3_VO_4_, proteinase inhibitor mixture, 0.4 mM PMSF) and lysed by sonication followed by centrifugation at 16,000 × g at 4 °C for 10 min. The supernatants (500 μg of total proteins) were used to incubate with anti-MFN2 antibody (Cell signaling #11925) or anti-Flag M2 affinity gel (Sigma, MO) for 2 hour at 4 °C. Then, protein G beads (Thermo Fisher Scientific, MA) were added and incubated for another 4 hrs. The immunoprecipitation complex were then washed twice with lysis buffer, boiled in 2× SDS-PAGE loading buffer and analyzed by Western blotting.

### Immunofluorescence Microscopy

MCF-7 cells were plated on the coverslips and fixed with 4% paraformaldehyde and washes with PBS. After permeabilization with 0.5% Triton X-100 in PBS and blocking with 10% goad serum, cells were incubated with and MitoTracker Red CMXRos (Cat: M7512) following manufacture instruction. Cells were then incubated with primary antibodies for MFN2 (1:100, cell signaling) and Rictor (1:50 Santa Cruz) at 4 °C overnight. Then, cells were washed 3 times with PBS and incubated with secondary fluorescent antibody at room temperature for 1 hr. After washing, cells on coverslips were mounted using prolong gold antifade mountant with DAPI (Thermo Fisher Scientific, MA) and analyzed under confocal microscope.

### Tumor xenograft model

Male BALB/c nude mice (5-week-old and weighing 17–18 g) were purchased from Shanghai Laboratory Animal Resource Center, and were maintained in a pathogen-free environment. Tumor model were generated by subcutaneously injection of 1 × 10^6^ cells. Tumor bearing mice were randomly grouped and tumors were allowed to grow to an average volume of 100 mm^3^ before treatment. Tumor volumes were measured by caliper once every 3days and calculated with the formula: V = (L × W^2^)/2 (L, length; W, width). For the P529 treatment, 20 mg/kg P529 was S.C. injected every 4 days for 24 days. Mice were sacrificed by inhaled CO_2_ at the end of treatment. Harvested tumors were weighed and immediately fixed in formalin for immunohistochemistry.

All proposals were approved and supervised by the institutional animal care and use committee of Putuo Hospital, Shanghai University of Traditional Chinese Medicine, P.R. China. All animal procedures complied with the National Institute of Health guidelines for the Care and Use of Laboratory Animals and were approved by the Institutional Animal Care and Use Committee.

### Immunohistochemistry

Tissues were fixed in 10% formalin, embedded in paraffin, and sectioned (5-mm thickness). For immunohistochemistry of MFN2, Ki67 and p-Akt (S473) was conducted as follows: slides were deparaffinised and incubated for 10 min with 3% H_2_O_2_ in water to quench the endogenous peroxidase activity. The heat induced antigen retrieval method was used for the detection of antigens. Tissues were incubated with 5% normal rabbit serum in TBS (0.05 M Tris-HCl, 0.5 M NaCl, pH = 7.4) for 30 min at room temperature. Dilution of the primary antibodies was as follows: 1:100 for mouse monoclonalanti-Ki67 (M7240, Dako Cytomation Denmark A/S, Glostrup, Denmark) and 1:200 for rabbit polyclonal anti-pAkt (Cell Signaling). The indirect avidin–biotin– peroxidase method was applied, using the appropriate secondary antibodies, for 30 min at room temperature. The EnVision (K4007, Dako) signal enhancement system was used to develop the bound antibodies. Sections were counterstained with Harris haematoxylin, dehydrated and mounted. For quantifications, 30 random images (400x) per experimental group were captured with a microscope (Leica, Wetzlar, Germany) equipped with the Analysis software.

### Patient survival assay

The Kaplan-Meier curve for lung or breast cancer patients with high or low MFN2 mRNA level was get from Kaplan-Meier Plotter (http://kmplot.com). For evaluation of the survival curve for patients with different MFN2 protein expression, breast tumor samples were collected at the time of surgical resection at Putuo Hospital, Shanghai University of Traditional Chinese Medicine, P.R. China, from January 2010 to December 2011. Written informed consent was obtained from the patients, in accordance with the institutional guidelines, before sample collection, and the study was approved by the Committees for the Ethical Review of Research at the Putuo Hospital, Shanghai University of Traditional Chinese Medicine, P.R. China. The methods were performed in accordance with the approved guidelines. The association of MFN2 expression with overall survival was analyzed by Kaplan-Meier Survival curve and Log-rank test.

### Statistical analysis

All data were derived from at least 3 independent experiments and results are expressed as mean ± standard error. Differences were assessed using the Student’s t-test or the Kruskal-Wallis test. P < 0.05 was deemed statistically significant.

## Result

### Low MFN2 expression predicts poor prognosis in cancer patients

**Figure 1:**
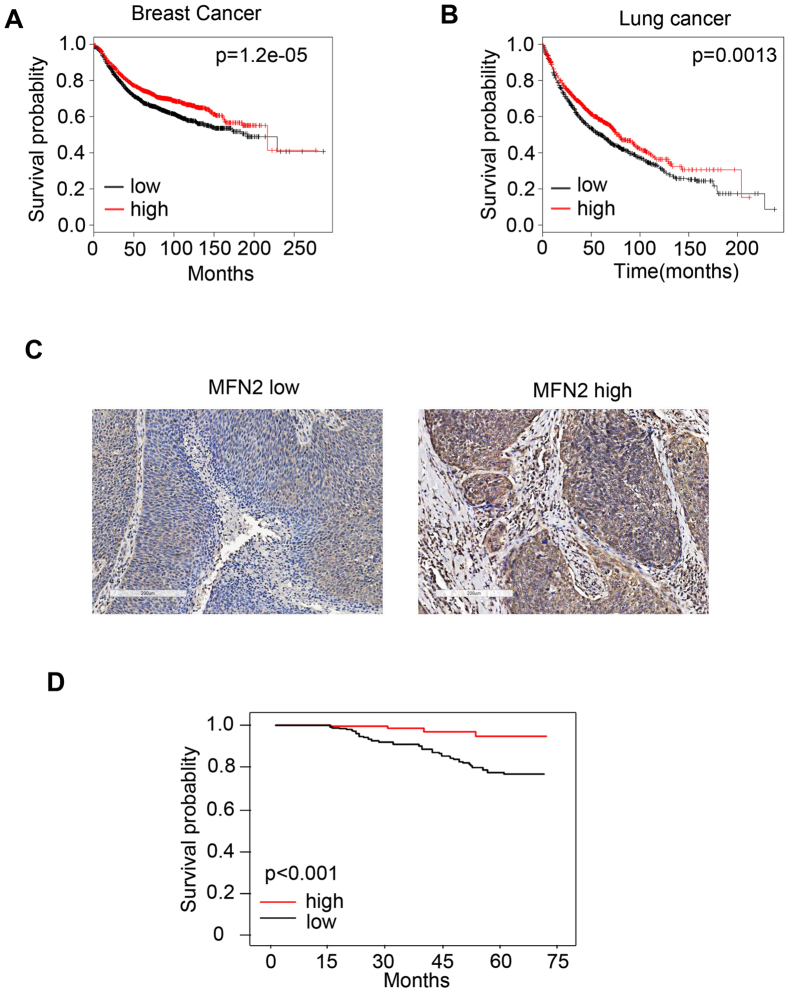
Cancer patients with MFN2 low expression are associated with poor prognosis. (**A**,**B**) The Kaplan-Meier curve for breast cancer (**A**) and non small lung cancer cell (NSLCC) (**B**) patients with high or low MFN2 mRNA level was get from Kaplan-Meier Plotter (http://kmplot.com). (Affy id/Gene symbol: 201155_s_at; Survival: RFS (n = 3955); Follow up threshold: all; and Auto select best cutoff and user selected probe set were selected to generate these figures). (**C**) Immunohistochemistry identifies the expression of MFN2 from breast cancer patients by experienced pathologist, representative pictures are shown. (**D**) Human breast cancer patient samples were collected at the time of surgical resection at Putuo Hospital, Shanghai University of Traditional Chinese Medicine, P.R. China, from January 2008 to December 2010. MFN2 expression level was then identified into MFN2 low group (n = 118) and MFN2 high group (n = 131) by experienced pathologist. Six year survival curves from patients of low and high MFN2 are shown.

To study the function of MFN2 in cancers, the Kaplan-Meier curves for lung or breast cancer patients with high or low MFN2 mRNA level were generated from Kaplan-Meier Plotter (http://kmplot.com). As shown in the Fig. 1A and B, both breast and lung cancer patients with low MFN2 expression were associated with poor prognosis as compared to patients with high expression of MFN2. To confirm these results, we collected human breast cancer patient samples at the time of surgical resection at the Putuo Hospital, Shanghai University of Traditional Chinese Medicine, P.R. China, from January 2008 to December 2010. MFN2 expression was then classified as MFN2 low group (n = 118) and MFN2 high group (n = 131) by experienced pathologist as shown in Fig. 1C. By using this set of data, Patient survival curve demonstrated that breast cancer patients with low MFN2 expression indeed had a poor prognosis, confirming that decreased levels of MFN2 were associated with poor prognosis in breast cancer patients (Fig. 1D).

### MFN2 loss enhances growth in breast cancer and lung cancer cells

**Figure 2:**
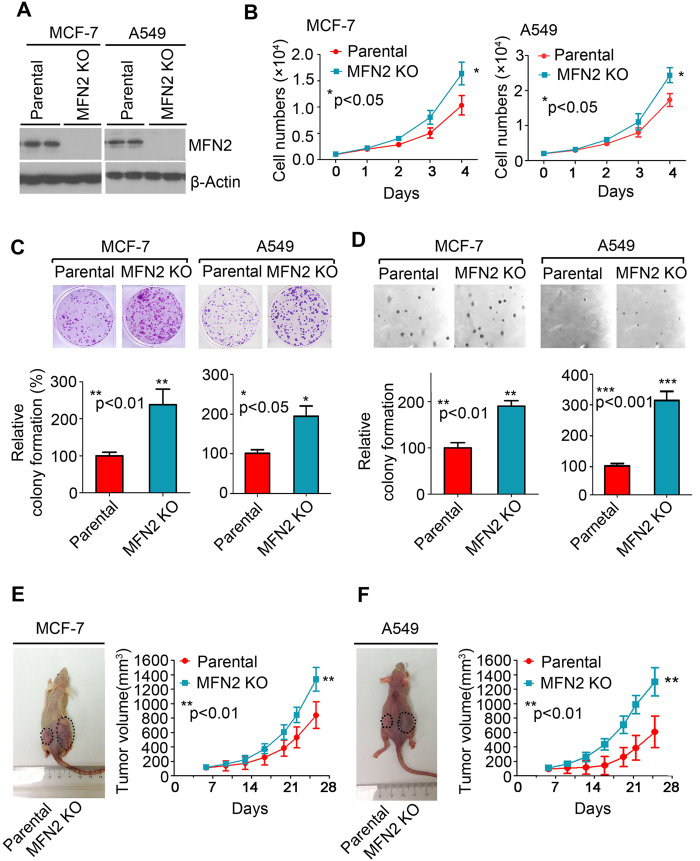
MFN2 loss enhances growth in breast cancer and lung cancer cells. (**A**) Western blot to test the efficiency and specificity of MFN2 knockout plasmid transfection was shown completely loss of MFN2 relative to parental MCF-7 and A549 cells. (**B**) Cell growth rate from parental and MFN2 knockout MCF-7 cells (left) and A549 cells (right) at indicated time point. (**C**,**D**) Colony formation (**C**) and soft-agar colony formation (**D**) assay of parental and MFN2 knockout MCF-7 and A549 cells. 800–1000 cells were plated in 6-well plate in complete medium and cultured for 10–14 days and colonies were stained and counted (**C**) and 10^4^ of cells were plated in complete medium containing 0.6% argose and cultured for 3 weeks and the colonies were stained and counted (**D**). (**E**,**F**) Tumor growth curve for MCF-7 (**E**) and A549 (**F**) xenogaft tumor models.

To investigate the effect of MFN2 in breast and lung cancer cells, MFN2 was knocked out by transfection of a Crispr/Cas9 KO plasmid in MCF-7, a breast cancer cell line, and A549, a lung cancer cell line. The efficiency and specificity of MFN2 knockout were shown to be of complete deletion relative to parental MCF-7 and A549 cells by Western blot analysis (Fig. 2A). Intriguingly, we observed that both MCF-7 and A459 cells with knockout of MFN2 grew significantly more rapidly than the parental cells (Fig. 2B), indicating a negative role of MFN2 in regulating cell growth. Consistently, frequencies of colony formation from MFN2 knockout MCF-7 and A459 cells were increased dramatically under both regular medium culture (Fig. 2C) and soft agar colony formation (Fig. 2D) conditions. We next examined the impact of MFN2 knockout on tumor growth *in vivo*. MFN2 deficient tumor cells from both MCF-7 and A549 exhibited significant stronger progression, as evaluated by larger tumor volume over the treatment period (Fig. 2E and F). Taken together, all these results suggest that MFN2 negatively regulates tumor growth and progression.

### MFN2 loss enhances cancer metastasis

**Figure 3:**
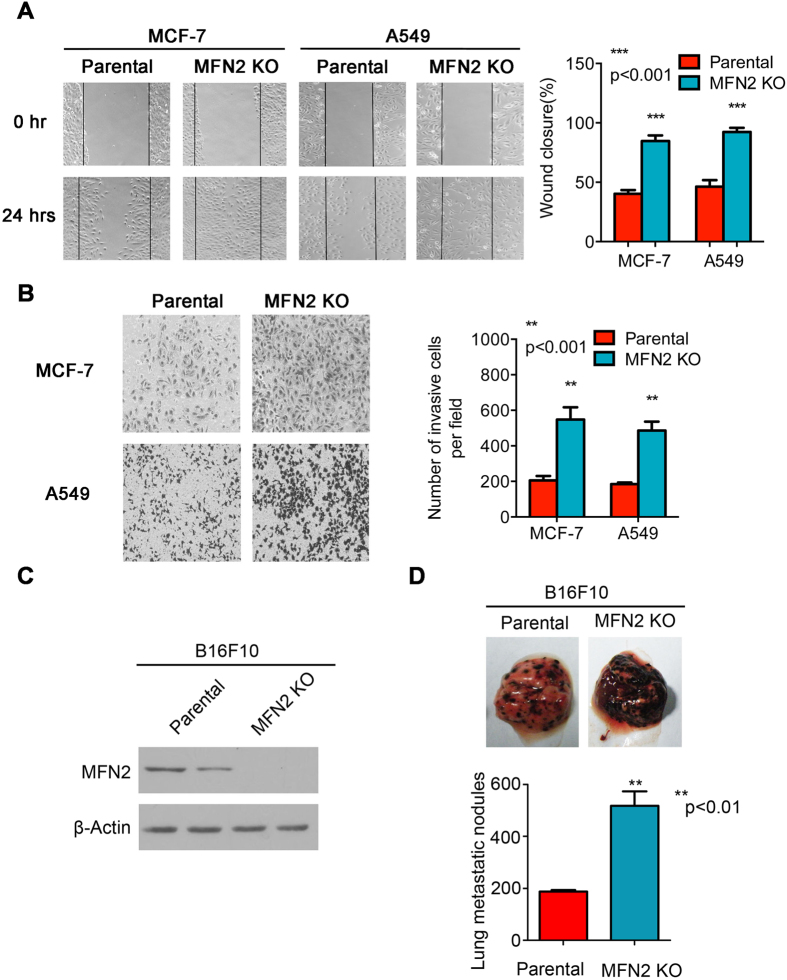
MFN2 loss enhances cancer metastasis. (**A**) *In vitro* wound healing assay with human MCF-7 and A549 cells after knock out with MFN2 expression. Image was acquired at 0, 24 h time points after scratching (left). Quantification of wound closure was calculated (right). (**B**) Representative staining of invasive potentials of human MCF-7 and A549 cells from *in vitro* transwells assay (left). Quantification of invasive cells per field was analyzed (right). Data were compared using the two-tailed Students *t*-test, **P < 0.01. (**C**) Immunoblotting for MFN2 and endogenous β-actin in parental and MFN2 knock out B16F10 cells. (**D**) MFN2 loss significant enhanced tumor metastasis in B16F10 melanoma lung metastasis model *in vivo*. (n = 10 mice per group).

Given that the invasive property of tumor cells contributes to poor prognosis, we next addressed whether MFN2 affects cell migration and invasion. Wound healing assay indicated that knock-out of MFN2 in both MCF-7 and A549 cells resulted in a significantly increased cell migration comparing with their parental cells (Fig. 3A, left panels). A quantitative analysis demonstrated that the wound closure rate was increased from 45% to 85% in both cells when MFN2 was knocked out (Fig. 3A, right panel). Consistently, we observed, in a transwell experiment, that more cells invaded into the bottom wells when MFN2 was knocked out (Fig. 3B). These results suggest that MFN2 regulates the abilities of migration and invasion for tumor cells *in vitro*. To further address whether MFN2 regulates the metastasis of tumor cells, we knocked out MFN2 in B16F10 melanoma cells (Fig. 3C), which showed high invasion ability *in vivo*. We then injected the cells into nude mice and observed the tumors in the lung. The results showed that knockout of MFN2 in the B16F10 melanoma cells significantly promoted metastasis in lung tissue at 14 days after injection (Fig. 3D), suggesting that MFN2 inhibits tumor migration and invasion *in vivo*.

### MFN2 directly interacts with Rictor and represses mTORC2/Akt signaling

**Figure 4:**
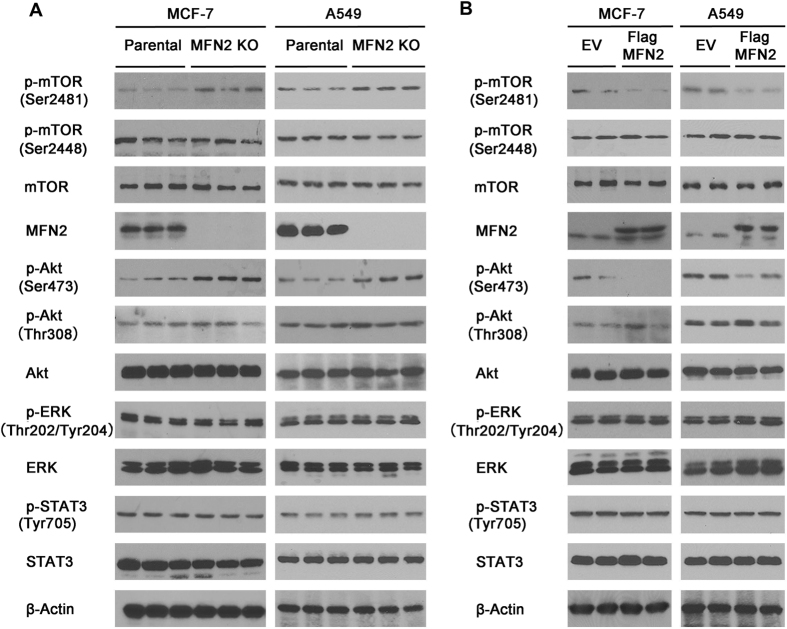
MFN2 represses mTORC2/Akt signaling pathway. (**A**) Whole cell lysates were prepared from parental and MFN2 knockout MCF-7 (left) and A549 (right) cells and examined for expression of MFN2 and Akt, mTOR, Erk, and Stat3 activities by immunoblotting with anti-phospho-Akt-pS473, anti-phospho-mTOR-S2481/S2448, anti-phospho-Erk, and anti-phospho-Stat3 antibodies. Blots were stripped and reprobed with anti-pan-Akt, anti-pan-mTOR-S2481/S2448, anti-pan-Erk, and anti-pan-Stat3 antibodies to check protein loading and β-Actin levels. Phosphorylation of Akt-S473 and mTORC2-S2481, but not Akt-S308, mTORC2-S2448, Erk, Stat3, is increased in MFN2 knockout MCF-7 and A549 cells. (**B**) Western blot analysis of the overexpressed MFN2 signaling pathway in MCF-7 and A549 cells. Phosphorylation of Akt-pS473 and mTORC2-pS2481, but not Akt-pS308, mTORC2-pS2448, Erk, Stat3, is inhibited by overexpression of MFN2.

To decipher the mechanism underlying the cell biological behavior in the absence of MFN2, we next analyzed the intracellular signaling in MCF-7 and A549 cells by Western blot. The results showed that knockout of MFN2 effectively promoted the phosphorylation of mTORC2-pS2481 while the total mTOR levels maintained unchanged (Fig. 4A). As mTORC2 was reported to phosphorylate Akt-pS473^29^,^30^, we examined the status of Akt, as well as ERK and STAT3, two critical pathways in regulation of tumor cell growth and invasion. Our data showed that deletion of MFN2 elevated the phosphorylation of Akt-S473, but not Akt-S308, Erk, and Stat3 (Fig. 4A, bottom panels). Reciprocally, we over-expressed MFN2 in both MCF-7 and A549 cells. The results showed that over-expression of MFN2 significantly inhibited the phosphorylation of Akt(S473) and mTOR(S2481) but failed to affect the phosphorylation of Akt(S308), mTOR(S2448), ERK and STAT3 (Fig. 4B). All these results suggest that MFN2 specifically inhibits the mTORC2/Akt-pS473 pathway.

**Figure 5:**
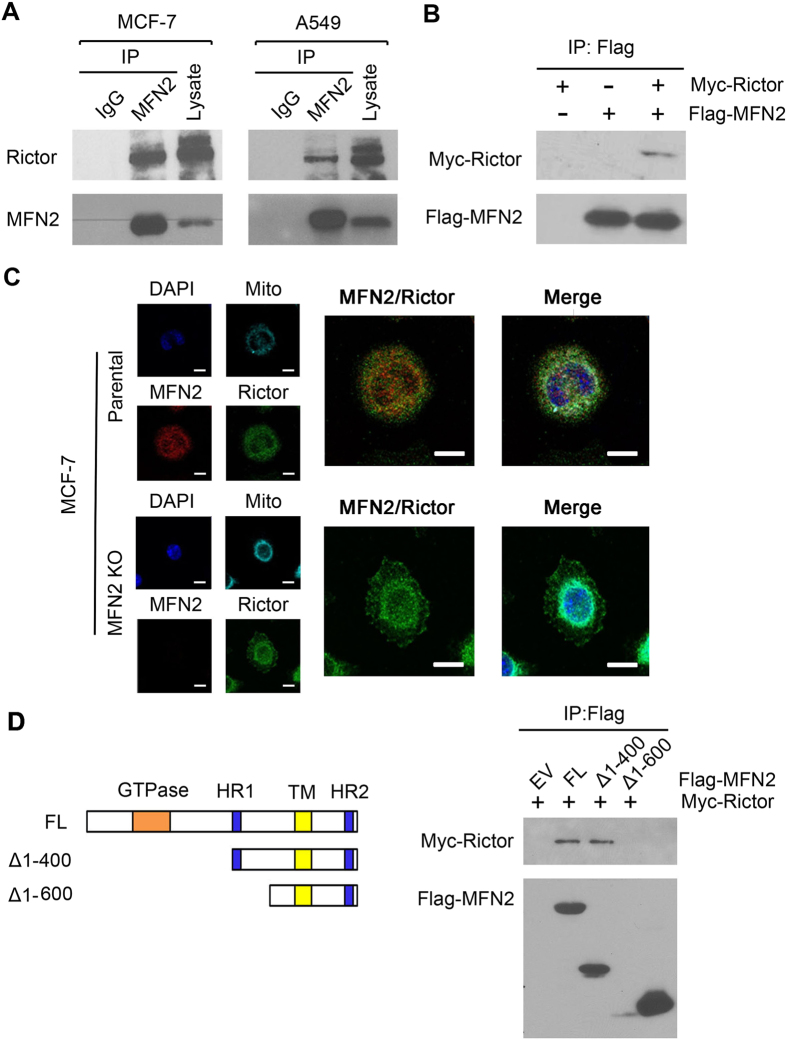
MFN2 directly interacts with Rictor. (**A**) Endogenous Co-immunoprecipitation of MFN2 and Rictor from MCF-7 and A549 cell extracts. (**B**) Immunoprecipitation between the tagged Flag-MFN2 and Myc-Rictor shown the interaction between Rictor and MFN2. (**C**) Immunofluorescent staining of MFN2 reflects overlapping with endogenous Rictor signal at mitochondria in parental MCF-7 cells. Scale bar = 5 μm. (**D**) HR1-HR2, HR2 containing constructs were generated by site-directed mutagenesis (see details in method). Δ1-600 construct containing only HR2 region fail to bind Rictor, while Δ1-400 construct containing HR1 and HR2 regions and full length of MFN2 directly interact with Rictor.

To investigate how MFN2 inhibits the mTORC2 activity, we questioned whether MFN2 directly interacts with any components of the mTORC2 complex. Immunoprecipitation experiment showed that MFN2 was associated with Rictor, a mTORC2 subunit, in both MCF-7 and A549 cells (Fig. 5A). To further validate the interaction between MFN2 and mTORC2, we performed co-immunoprecipitation assay using Flag-MFN2 and Myc-Rictor. The result showed that Flag-MFN2 and Myc-Rictor interacted in MCF-7 cells, confirming the interaction between Rictor and MFN2 (Fig. 5B). To demonstrate the physical interaction, we performed microcopy co-localization analyses to characterize the subcellular localization of protein of MFN2 and mTORC2. The results showed that both MFN2 and mTORC2 signal overlapped with mitochondria in the MCF-7 cells (Fig. 5C) and A549 cells (data not shown), supporting the notion that MFN2 plays a role through regulation of mTORC2 pathway (Fig. 5C). All the results suggest that MFN2 plays a role in the regulation of mTORC2 pathway through interaction with Rictor.

### MFN2 binding on Rictor is required for the inhibition of mTORC2 signaling

**Figure 6:**
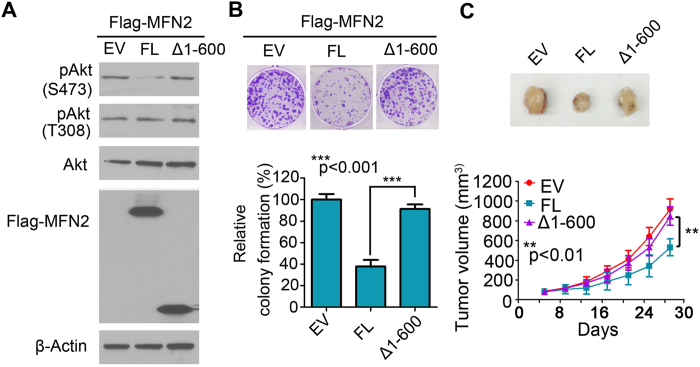
MFN2 binding on Rictor is required for inhibition of mTORC2 signaling. (**A**) MFN2-Δ1-600 constructs containing only HR2 regions failed to inhibit Akt-pS473. (**B**) Representative pictures shown that MFN2-Δ1-600 constructs did not inhibit colony formation. Quantification of relative colony formation from different groups were analyzed (down). (**C**) Representative pictures shown tumor increased tumor volume after transfection of MFN2-Δ1-600 constructs (up). Statistics analysis of tumor volume at indicated time points after injection *in vivo* (down).

Since MFN2 is composed of a conserved molecular structure containing the N-terminal GTPase domain, two regions of hydrophobic heptad-repeat coiled-coil motifs, HR1 and HR2, and two tandem transmembrane domains near the C-terminus^31^,^32^ (Fig. 5D). We mapped the binding site of MFN2 on Rictor using site-directed mutagenesis analysis. The domains tested in the analysis include Δ1-400 construct containing both HR1 and HR2 regions, Δ1-600 construct containing only HR2 region, and full-length MFN2 (Fig. 5D). We co-transfected with flag tagged MFN2 full length (FL) or deletion mutants and myc tagged Rictor, and performed co-immunoprecipitation in cultured. As shown in Fig. 5D, Myc-Rictor was detected in the anti-Flag immune complex along with HR1-HR2, and full length MFN2, but not in Δ1-600 construct group, indicating constructs containing HR1 interact with mTORC2 (Fig. 5D). To further define whether the interaction of the region of HR1 is required for the activation of mTORC2, pAkt signaling was examined after transfection of full length MFN2 or indicating deletion mutants. It showed that Δ1-600 transfected cells failed to decrease expression of pAkt-S473 as compared to full-length MFN2, suggesting MFN2 and mTORC2 interaction is required for the inhibition of Akt (Fig. 6A). Functional analysis that colony formation from Δ1-600 construct failed to inhibit colony formation (Fig. 6B). Moreover, to rule out other possible mechanisms other than Rictor play a role in the failure of inhibitory colony formation of by Δ1-600 construct, we treated FL and truncated MFN2 (Δ1-600) groups with or without Rictor siRNA. We then monitored colony formation from each group and found that MFN2 (Δ1-600) treated with Rictor siRNA could significantly reduce colony formation as compared to MFN2 (Δ1-600) treated with scramble siRNA group, which rule out other possible mechanisms other than Rictor (Supplementary Fig. 1). To confirm the consequence of Δ1-600 construct *in vivo*, we injected MCF-7 cells transfected with Δ1-600 construct, full length MFN2 and EV construct into mice. Indeed, tumor volume at indicated time points showed that Δ1-600 construct failed to inhibit tumor growth *in vivo*, strongly supporting the hypothesis that MFN2 binding on Rictor is required for the inhibition of mTORC2 signaling (Fig. 6C).

### Inhibition of mTOCR2 significantly suppresses MFN2 deficient tumor growth

**Figure 7:**
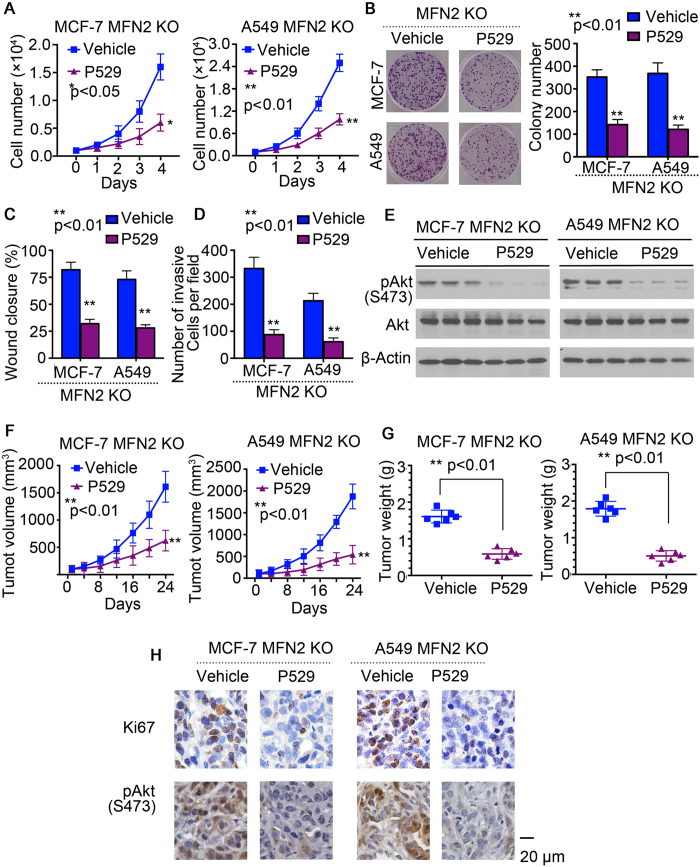
Inhibition of mTOCR2 significantly suppress MFN2 deficient tumor growth. (**A**) Cell growth rate fromP529 (2 μM) and vehicle treated MFN2 knockout MCF-7 (left) and A549 cells (right). (**B**) Representative pictures (left) and statistical analysis (right) shown colony formation from P529 and vehicle treated MFN2 knockout MCF-7 and A549 cells. (**C**,**D**) *In vitro* wound closure of P529 and vehicle treated MFN-2 knockout MCF-7 cells (**C**) and A549 cells (**D**) from 24 h after scratch assay. (**E**) Whole cell lysates were prepared from P529 and vehicle treated MFN2 knockout MCF-7 (left) and A549 (right) cells and examined for Akt activities by immunoblotting with anti-phospho-Akt-pS473 antibodies. Blots were stripped and reprobed with anti-pan-Akt antibodies to check protein loading and β-Actin levels. (**F**,**G**) Mice were injected with MFN-2 knockout MCF-7 cells and A549 cells and then were treated with P529 and vehicle (n = 6). Tumor volume (**F**) and tumor weight (**G**) were analyzed at indicated time point. (**H**) Histochemistry staining of Ki67 and p-Akt shown increased positive cells from P529 treated tumors originally from injection of MFN2 knockout MCF-7 and A549 cells compare to vehicle-treated group.

To ensure that the above approach represented a reliable readout and to explore the impact of blocking mTORC2 on MFN2 knockout cells, we next treated MFN2 knockout cells with P529, a known inhibitor of mTORC1/mTORC2. As expected, cell growth rate and colony formation from P529-treated groups were significantly deceased compared with vehicle groups from MFN2 knockout MCF-7 and A549 cells (Fig. 7A,B). Wound closure from scratch assay also showed that P529-treated group displayed significantly less invasion from MFN2 knockout MCF-7 and A549 cells *in vitro* through dephosphorylation of Akt-pS473 (Fig. 7C–E). In addition, to see the suppression effect of mTORC2 inhibitor in WT MFN2 cells, we treated both MCF-7 and A549 without MFN2 knockout cell lines, and found treatment of mTORC2 inhibition in MFN2 normal groups did not significantly reduce colony formation (Supplementary Data Fig. 2), indicating suppression of mTORC2 was significantly effected in MFN2 deficient tumors. We next evaluated the tumor characterization by inhibition of mTORC2 from MFN2 knockout MCF-7 and A549 cells *in vivo*. As shown in Fig. 7F–H, tumor volume and tumor weight were significantly decreased from in MFN2 knockout MCF-7 and A549 cells with administration of P529 through inhibition of cell cycle and Akt pathway.

## Discussion

There is accumulating evidence that MFN2 influences tumor growth and progression, however, the mechanism(s) by which this occurs remains unclear. We show that MFN2 is physically associated with mTORC2, and MFN2 mediates mTOCR2-Akt signaling function. MFN2 localizes to mTORC2 where it phosphorylates and activates Akt.

mTORC2 is critically involved in various cellular and developmental processes, but knowledge of its regulations is limited. Our studies have identified MFN2 as a direct mTORC2 inhibitor in cancer cell lines *in vitro* and *in vivo*. MFN2 inhibits the kinase activity of mTORC2 toward Akt^33^,^34^. This function of MFN2 is mediated by its direct interaction with mTORC2, revealing a canonical role of MFN2 regulated mTORC2 signaling pathway. Furthermore, we show that MFN2 negatively regulates cell survival through inhibiting mTORC2 and Akt^S437^, validating the biological significance of this newly discovered regulatory mechanism.

Akt regulates many physiological processes in addition to cell survival and proliferation, including glucose metabolism and cellular differentiation. Regulation of Akt by MFN2 in those processes warrants future investigation. Akt, also known as a proto-oncogene, is involved in tumorigenesis by promoting the proliferation, survival, and metastasis of cancer cells^35^. Evidence has shown a direct role of mTORC2, at least partly through Akt, in driving tumorigenesis^36^. Future studies of a role of MFN2 in tumor suppression, especially in the context of activation Akt, may prove informative for the understanding and therapeutic strategy against cancer.

mTOR is a highly conserved protein kinase and plays a central role in many types of cancer progression^37^. To validate the role of mTORC2 in mediating the pathogenic effects of MFN2 knockout MCF-7 and A549 cells. We treated MFN2 knockout MCF-7 and A549 cells, which developed profound tumor with severe invasion (Figs 2 and 3D), with mTORC1/2 inhibitor P529. As shown in Fig. 7, treatment with P529 for 24 days effectively reduced tumor growth. The robust therapeutic effects are correlated with the reduction of hyperactivation of cell cycle (Fig. 7H).

In summary, our *in vitro* and *in vivo* studies have demonstrated the MFN2-knockout MCF-7 and A549 cells have profound pathogenic effects on tumor progression. Elevated mTORC2 expression in MFN2 knockout MCF-7 and A549 cells appears to play a major role in mediating tumor growth and progression. This study reveals the critical contribution of MFN2 to suppress cancer progression through inhibition of mTORC2/Akt signaling and identifies mTORC2 as a potential therapeutic target for controlling tumor aggression.

## Additional Information

**How to cite this article:** Xu, K. *et al*. MFN2 suppresses cancer progression through inhibition of mTORC2/Akt signaling. *Sci. Rep.* **7**, 41718; doi: 10.1038/srep41718 (2017).

**Publisher's note:** Springer Nature remains neutral with regard to jurisdictional claims in published maps and institutional affiliations.

## Supplementary Material

Supplementary Information
