# Supplementary material for: MFN2 suppresses cancer progression through inhibition of mTORC2/Akt signaling
Source: Sci Rep. 2017 Feb 8;7:41718. doi: 10.1038/srep41718 (PMC5296837; doi:10.1038/srep41718)

MFN2 suppresses cancer progression through inhibition of mTORC2/Akt signaling

Ke Xu[1#], Guo Chen[2#], Xiaobo Li[3], Xiaoqin Wu[4], Zhijie Chang[5], Jianhua Xu[1], Yu Zhu[6], Peihao Yin[1], Xin Liang[7], Lei Dong[2,8]

[1] Central laboratory, General Surgery, Putuo Hospital, and Interventional Cancer Institute of Chinese Integrative Medicine, Shanghai University of Traditional Chinese Medicine, 164 Lanxi Rd, Shanghai 200062, PR China.

[2] Department of Radiation Oncology, Emory University School of Medicine, Atlanta, GA 30322, USA.

[3] Tianjin Key Laboratory of Molecular Design and Drug Discovery, Tianjin Institute of Pharmaceutical Research, Tianjin 300193, China.

[4] School of Pharmacy, Institute for Liver Diseases of Anhui Medical University, ILDAMU, Key Laboratory of Anti-inflammatory and Immune Medicine, Anhui Medical University, Hefei, China, 230032

[5] State Key Laboratory of Biomembrane and Membrane Biotechnology, School of Medicine, School of Life Sciences, Tsinghua University, Beijing 100084, China

[6] Department of Clinical Laboratory, Tianjin Huanhu Hospital, Tianjin Key Laboratory of Cerebral Vessels and Neural Degeneration, Tianjin 300350, China.

[7] State Key Laboratory of Bioreactor Engineering & Shanghai Key Laboratory of New drug design, School of pharmacy, East China University of Science and Technology, 130 Meilong Rd, Shanghai 200237, PR China.

[8] Department of Pediatrics, Division of Hematology/Oncology, Aflac Cancer and Blood Disorders Center, Children's Healthcare of Atlanta, Emory University School of Medicine, Atlanta, GA 30322, USA.

[#]Ke Xu and Guo Chen contributed equally to this work

Correspondence should be addressed to:

Lei Dong, M.D., Ph.D.

Department of Pediatrics
Division of Hematology/Oncology
Aflac Cancer and Blood Disorders Center
Emory University School of Medicine
1760 Haygood Drive NE, HSRB E363
Atlanta, GA 30322
Phone: +1 6462880069
Email: ldong8@emory.edu; dlei4395@yahoo.com

Ke Xu, Ph.D.

Central laboratory of Putuo Hospital
Interventional Cancer Institute of Chinese Integrative Medicine
Shanghai University of Traditional Chinese Medicine
164 Lanxi Rd
Shanghai 200062, PR China
Phone: +86 13818524869
Email: cola519@163.com

Xin Liang, Ph.D.

State Key Laboratory of Bioreactor Engineering & Shanghai Key Laboratory of New drug design,
School of pharmacy,
East China University of Science and Technology,
130 Meilong Rd,
Shanghai 200237, PR China
Phone: +86 13681683436
Email: xin.liang@ecust.edu.cn

Key words: MFN2, mTORC2, lung cancer, breast cancer, tumor

Supplementary Data Figure 1. Rictor siRNA suppress colony formation in truncated MFN2

Supplementary Data Figure 2. P529 treatment in MFN2 WT MCF-7 and A549 cell lines

Supplementary Data Figure 1

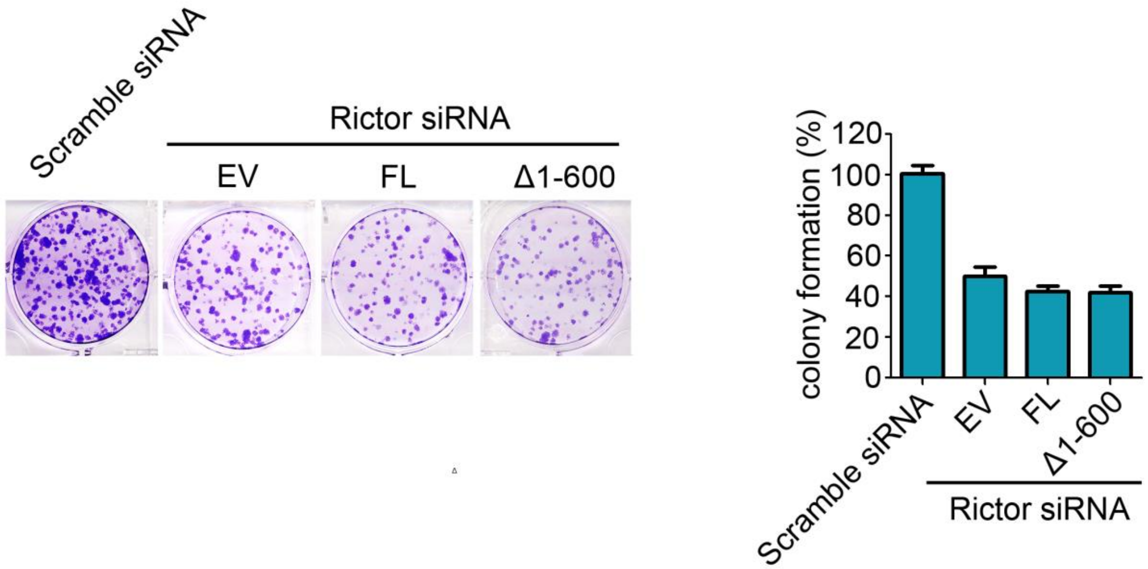

Supplementary Data Figure 2

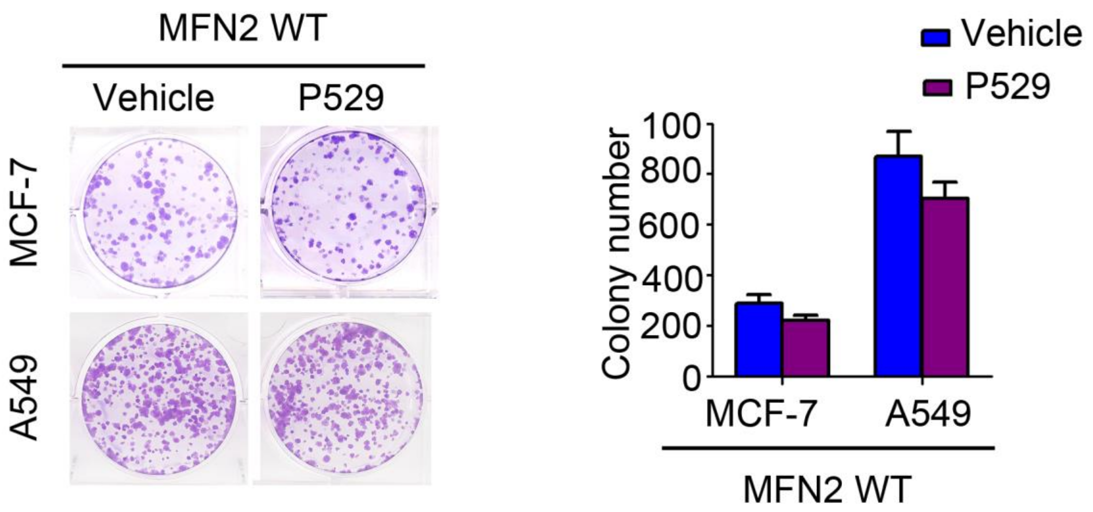

Supplement: Supplementary Information [file srep41718-s1.pdf]
